# Delayed Stroke following Blunt Neck Trauma: A Case Illustration with Recommendations for Diagnosis and Treatment

**DOI:** 10.1155/2017/3931985

**Published:** 2017-02-09

**Authors:** Best Anyama, Daniela Treitl, Jeffery Wessell, Rachele Solomon, Andrew A. Rosenthal

**Affiliations:** ^1^Mount Sinai Medical Center, 4300 Alton Road, Miami Beach, FL 33140, USA; ^2^Memorial Regional Hospital, Division of Acute Care Surgery and Trauma, 3501 Johnson Street, Hollywood, FL 33021, USA; ^3^Nova Southeastern University, College of Osteopathic Medicine, 3301 College Avenue, Fort Lauderdale, FL 33314, USA

## Abstract

Blunt cerebrovascular injury (BCVI) to the carotid artery is a relatively rare injury that is difficult to identify even with imaging. Any symptoms or neurological deficits following blunt neck injury mandate evaluation and consideration of BCVI. In an effort to highlight this issue, we report the case of a 31-year-old male patient who presented with left-sided weakness consistent with transient ischemic attack (TIA) and concussion. The patient's symptoms occurred within 24 hours of a blunt neck injury sustained by a knee strike during a basketball game. An initial computerized tomography (CT) scan of the brain was normal; a CT angiogram (CTA) of the neck and carotids did not reveal obstruction, dissection, stenosis, or abnormalities of the carotid or vertebral vessels and the patient was subsequently discharged. A magnetic resonance imaging (MRI) of the brain obtained four days after the initial injury demonstrated an acute infarct in the right middle cerebral artery (MCA) territory. Thus, despite initial negative imaging, neurological deficits must be aggressively pursued in order to prevent stroke in BCVI cases.

## 1. Introduction

Blunt cerebrovascular injury (BCVI) is relatively rare, with an incidence rate of 0.39–1.11% [1–3]. However, despite the rarity of BCVI, there is a high rate of morbidity associated with BCVI occurrence, leading to stroke in 10–20% of patients [4]. BCVI patients may present with an expanding hematoma (neck, nose, mouth, and ears), cervical bruit, focal neurological deficits, transient ischemic attacks (TIA), Horner's syndrome, or evidence of cerebral ischemic infarction [2, 5]. In the presence of stroke-like symptoms, a comprehensive history, clinical examination, and directed imaging following stroke guidelines are required. Left untreated, BCVI has been linked to increased risk of stroke (23–50%), morbidity (10–48%), and mortality (11–25%) [1–3, 6, 7]. To raise awareness regarding the diagnostic challenges associated with BCVI, we present a case of blunt neck trauma leading to a cerebral infarct in an adult male and discuss the clinical presentation, radiographic findings, and management.

## 2. Case Report

**Figure 1:**
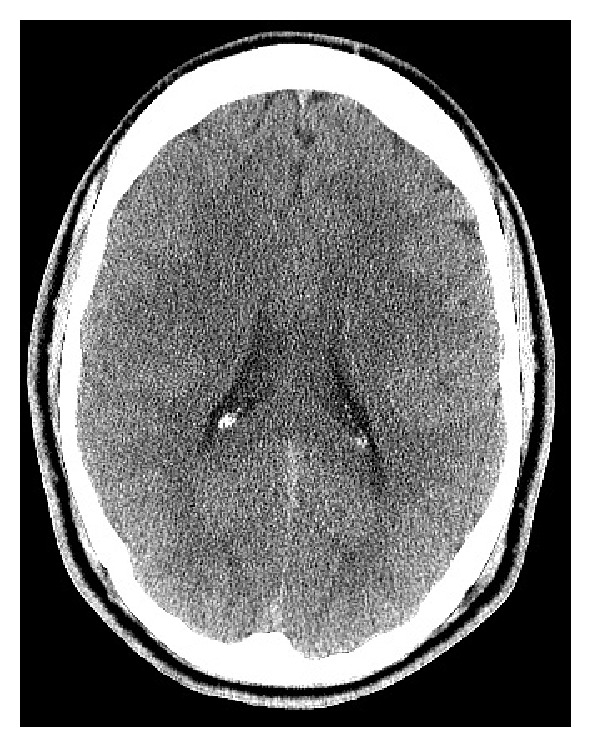
CT of the brain without contrast shows no intracranial abnormalities.

**Figure 2:**
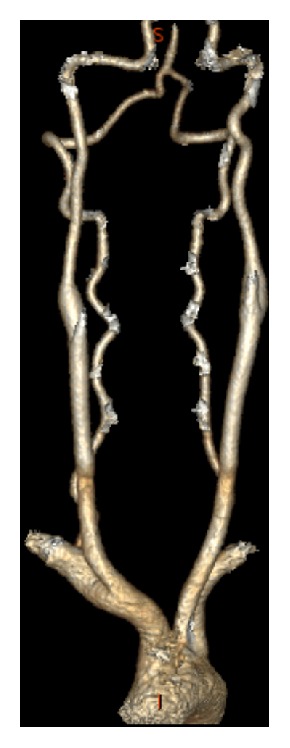
CT angiogram reconstruction of the neck shows no obstruction, dissection, stenosis, or abnormalities.

A 31-year-old male with no significant history presented to the emergency department (ED) with left arm and leg weakness accompanied by headache and right-sided neck pain. The day prior while playing basketball, he sustained a blow to the right lateral neck during a collision with the knee of another player. That night, the patient had left arm and leg weakness accompanied by a facial droop, the latter resolving prior to presentation to the ED. In the ED, the patient had localized tenderness over the paracervical muscles and residual left arm and leg weakness. He had no carotid bruit, and the remainder of his physical exam was unremarkable. A brain computerized tomography (CT) scan without contrast (Figure 1) and CT angiogram (CTA) of the neck (Figure 2) were normal as read by an experienced neuroradiologist. His echocardiogram was also normal. He was diagnosed with a concussion and possible cervical radiculopathy and discharged with cyclobenzaprine, methylprednisolone, and analgesics.

**Figure 3:**
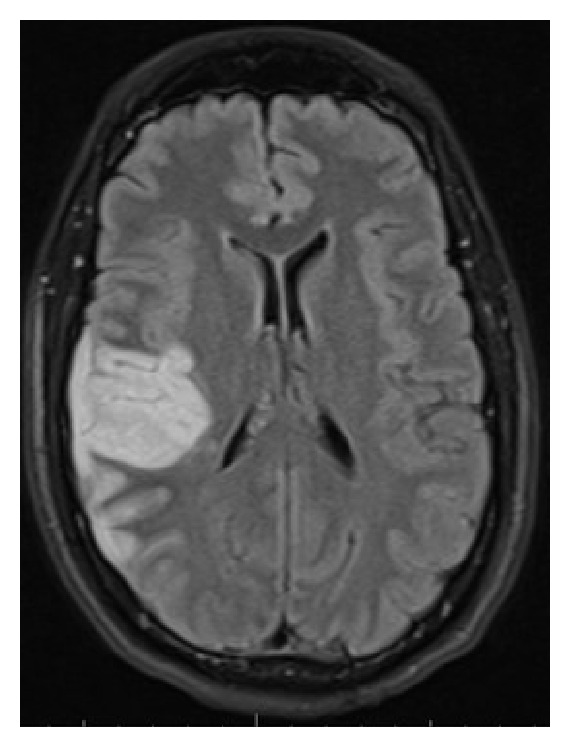
MRI of the brain with acute ischemic infarct in the right lobe.

Four days after the injury occurred, the patient presented with persistent dizziness, right-sided headache, and left-sided weakness. During the physical exam a left-sided pronator drift was noted. Magnetic resonance imaging (MRI) of the brain demonstrated an acute ischemic infarct in the right frontoparietal lobe, superior aspect of posterior right temporal lobe, and posterior aspect of the right insular cortex with a 4 mm right-to-left midline shift (Figure 3). MRI of the neck showed no evidence of cervical vertebral body fractures. Bilateral carotid ultrasound and CTA of the brain and neck including the skull base were negative for dissection and stenosis of the carotid bulbs and vertebral arteries, respectively. Aspirin therapy was initiated and the patient was discharged home with outpatient rehabilitation one week later. During follow-up, five months after injury, the patient continued to have intermittent left arm numbness and headaches along with comprehension difficulty, intermittent confusion, and trouble sleeping.

## 3. Discussion

Blunt trauma to the neck with associated BCVI can have devastating complications. Mechanisms in BCVI are thought to include cervical hyperextension or hyperflexion, internal carotid artery stretching over vertebral bodies one and two, direct cervical trauma, intraoral trauma, and basilar skull fracture involving the carotid canal [1, 5]. These mechanisms can lead to tearing of the intima, which creates a local thrombogenic area within the lumen. This area has the potential to dissect or form a pseudoaneurysm [4]. A severe occlusive dissection or embolization of the thrombus is a major risk factor for cerebral infarction [4, 5, 8].

Stroke caused by BCVI occurs within the first 24 hours 25–50% of the time [4]. Our patient's neurological deficits occurred within this time frame, despite having an unremarkable CT scan. Research supports that cerebral infarction may not be observed by CT scan until 24–48 hours after the cerebrovascular accident. However, using an MRI with diffusion-weighted sequences would allow this observation within minutes of the event [5]. In this case, a delayed MRI of the brain was obtained and demonstrated an acute cerebral ischemic infarction in the right middle cerebral artery territory.

Imaging modalities to diagnose BCVI include Duplex ultrasound, CTA, MRI, magnetic resonance angiography (MRA), and digital subtractive angiography (DSA). Thorough evaluation for BCVI should be performed in the presence of a clinical examination consistent with stroke, evidence of neck trauma, such as ecchymosis or bruits, and radiographic demonstration of neck injury, such as cervical spine fracture. The Denver Screening Criteria (DSC) was created from DSA studies to categorize the extent of arterial vessel wall injury in BCVI, defined as injury to the carotid or vertebral arteries caused by blunt force trauma [5]. Cerebrovascular injuries are divided into five categories with grades ranging from one to five [2, 5]. Grades 1 and 2 are low grade with less than and greater than 25% luminal stenosis, respectively. Grade 3 is defined by pseudoaneurysm and grade 4 by complete occlusion, while grade 5 is defined by free extravasation [9]. Despite having an established grading system, BCVI can be difficult to diagnose on imaging.

Generally, DSA is considered the “gold standard” for BCVI detection; however it is an invasive procedure with inherent risks [5]. Alternatively, both CTA and MRI have a high specificity but a low sensitivity for BCVI, with MRA being less optimal due to the timeframe required to complete the study [1]. In this particular case, an early MRI of both the brain and neck with a focus on vascular and spinal cord anatomy would have provided a more complete evaluation to explain the patient's presentation and objective neurologic symptoms. Support for or suspicion of cervical radiculopathy would have been confirmed during early MRI. A recent systematic review and meta-analysis showed that CTA scan sensitivity in detecting BCVI of all grades remains less than 80% when scans were read by a neuroradiologist using at least a 16-slice scanner and has a false negative rate of 79% for injuries classified as grade 2 or higher [10–12]. CTA scans may miss grade 1 BCVI; therefore DSA should be considered in patients with a moderate to high pretest probability, as measured with BCVI screening protocols [10].

In the case of our patient, there was no radiologic evidence of cerebral infarction until four days after injury when he presented with a stroke. CTA of the carotid arteries both during initial and follow-up scans were normal. Considering the presenting symptoms and negative CTA, MRI of the brain and cerebral vessels, echocardiogram, and observation would have been an appropriate approach to prompt further diagnostic testing with DSA [2, 4, 8, 10]. Considering the patient's initial cerebrovascular signs and symptoms, a stroke specialist or neurologist would have been an appropriate consultant despite negative imaging. Ultimately it was difficult to determine whether the stroke was secondary to an embolic phenomenon, thrombus, vasospasm, or low flow state.

To prevent thrombosis, embolization, and cerebral intracranial vessel clot propagation, the current initial recommendation for the management of BCVI is antithrombotic therapy [2]. Antiplatelet therapy reduces the risk of arterial thrombosis formation, which can help to prevent a cerebral infarction [3]. In addition, anticoagulation therapy with heparin has been associated with neurological lateralization deficit improvement [3, 4]. Research supports that antiplatelet and anticoagulation therapies have equivalent outcomes [6, 7]. Our patient underwent dual antiplatelet therapy with aspirin and clopidogrel after having a stroke four days after blunt neck trauma. Five months after the onset of symptoms, the patient remained stable with residual intermittent numbness but no further weakness.

## 4. Conclusion

Making the diagnosis of cerebral ischemia caused by BCVI requires a high index of suspicion. In patients with neurological deficits, continued work-up is necessary. Even in the presence of negative CT and CTA imaging, MRI of the brain and cerebral vessels should be considered when symptoms persist. Patients with a history or presentation consistent with TIA should be admitted and thoroughly evaluated to ensure timely diagnosis and treatment.
